# Complete genome sequence of *Stenotrophomonas maltophilia* ESTM1D_MKCAZ16_6a (Sm18), a Mexican environmental multidrug- and amoeba-resistant strain

**DOI:** 10.1128/mra.01304-25

**Published:** 2026-03-05

**Authors:** Pablo Vinuesa, Fulvia-Stefany Argueta-Zepeda, Julio César Valerdi-Negreros, Diana Marisol Vázquez-Enciso, Mauricio Daniel Osorio-Herrera, Daniela Gutiérrez-Hernández, Karina Mendoza-Gómez, Javier Rivera, Luz Edith Ochoa-Sánchez

**Affiliations:** 1 Centro de Ciencias Genómicas, Universidad Nacional Autónoma de México (UNAM) 61740 , Cuernavaca, Morelos, Mexico; 2 Programa de Maestría y Doctorado en Ciencias Bioquímicas, Universidad Nacional Autónoma de México 7180, Mexico City, Mexico; 3 Programa de Doctorado en Ciencias Biomédicas, Universidad Nacional Autónoma de México 7180, Mexico City, Mexico; 4 Instituto de Biotecnología, Universidad Nacional Autónoma de México 42560 , Cuernavaca, Morelos, Mexico; 5 Facultad de Ciencias, Universidad Nacional Autónoma de México 7180, Mexico City, Mexico; 6 Universidad Autónoma del Estado de Hidalgo 27781, Pachuca, Mexico; 7 Hospital Universitario 12 de Octubre 16473, Madrid, Spain; University of Southern California, Los Angeles, California, USA

**Keywords:** *Stenotrophomonas*, *Acanthamoeba*, metallostasis, multidrug resistance, amoeba, Mexico

## Abstract

*Stenotrophomonas maltophilia* ESTM1D_MKCAZ16_6a (Sm18) is a multidrug-resistant strain isolated from river sediments in Morelos, Mexico. It is capable of replicating within acid Rab7A-positive *Acanthamoeba castellanii* phagosomes. The complete genome was assembled from Oxford Nanopore and paired-end Illumina reads, encoding 3,970 proteins and 91 RNAs on a single circular 4.48 Mbp chromosome.

## ANNOUNCEMENT

*Stenotrophomonas maltophilia* ESTM1D_MKCAZ16_6a (Sm18) was isolated on MacConkey plates amended with 16 µg/mL ceftazidime from the sediment samples collected from the Las Estacas River (18.73262 N 99.11343 W), Morelos, Mexico (1). Sm18 was the only *S. maltophilia* strain found among the 108 environmental *Stenotrophomonas* isolates analyzed in that study (1). Strain Sm18 is multidrug-resistant, expressing metallo-β-lactamase activity, and resistant to 13 of the 15 drugs from five families tested (1), according to the 26th edition of the Clinical and Laboratory Standards Institute (2). Sm18 requires the manganese importer MntH to replicate efficiently within the acidified Rab7A-positive phagosomes of *Acanthamoeba castellanii* trophozoites (3, 4). We selected Sm18, a rare example of a well-characterized environmental *S. maltophilia* strain, as our experimental model to study the genes involved in metallostasis, virulence, and host adaptation.

Genomic DNA was extracted from a 5 mL overnight culture of strain Sm18 grown in LB at 30°C using Genomic-tips 100/G (Qiagen). All subsequent protocols were performed according to the manufacturer’s instructions. A short insert library was prepared using the Illumina Nextera XT DNA library preparation kit, followed by sequencing (2 × 300 cycles) on a MiSeq instrument (Illumina, Inc., San Diego, CA, USA). Raw fastq reads (4,054,325) were processed using Fastp version 0.20.0 (5), yielding 3,504,906 filtered paired-end reads. Long-read sequencing was performed on the same DNA preparation without DNA fragmentation or size fractionation using the Rapid Barcoding gDNA Sequencing Kit (SQK-RBK004), followed by sequencing on a MinION R9.4.1 device (Oxford Nanopore Technologies [ONT], Inc., Cambridge, MA, USA). All subsequent data analyses were performed using the software defaults. Base calling was performed using ONT’s Albacore Sequencing Pipeline (version 2.3.3), yielding 3.25 Gbp with an *N*_50_ of 11.8 kbp after filtering with Filtlong version 0.2.0 (6). Long read assemblies were generated using the TryCycler version 0.5.3 pipeline (7) with Miniasm version 0.2 (8)/Minipolish version 0.1.3 (9), Flye version 2.9 (10), and Raven version 1.8.1 (11) assemblers, followed by sequential polishing of the single consensus contig with the Illumina short reads using Medaka version 1.8.1 (12) and Polypolish version 0.5.0 (13). Circularity was confirmed with TryCycler.

**Fig 1:**
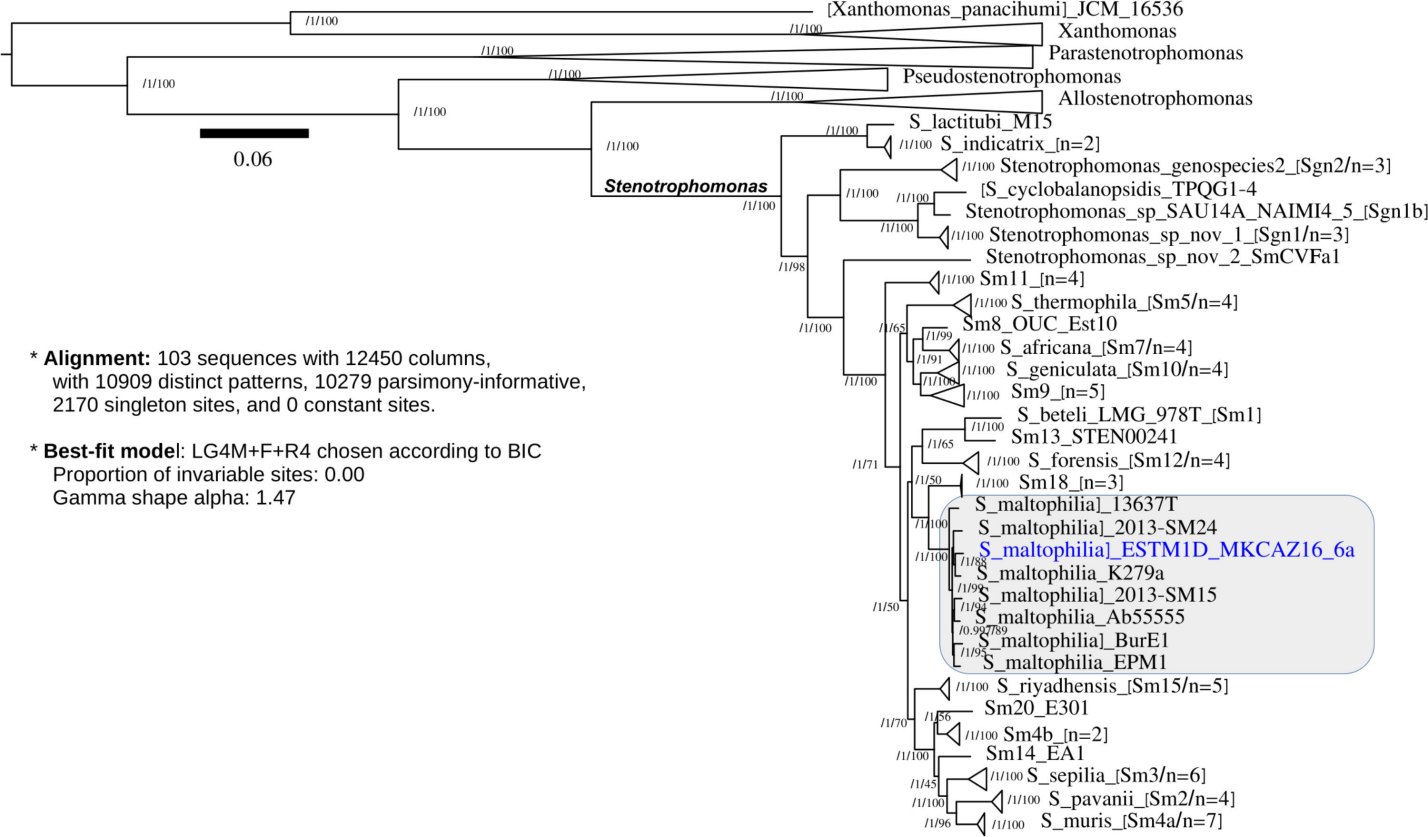
Maximum likelihood phylogeny estimated from a supermatrix of 106 core genome proteins selected by GET_PHYLOMARKERS out of 606 input core-genome sequences identified with GET_HOMOLOGUES from 103 genomes, showing that ESTM1D_MKCAZ16_6a (Sm18) is a *bona fide Stenotrophomonas maltophilia* strain, clustering in a perfectly supported clade with the type strain ATCC 13637^T^ and other well-characterized members of the species, such as K279a (16, 18). The tree was rooted with *Xanthomonas* spp. sequences. Numbers on bipartitions are aBayes/UFBoot support values. Validly described *Stenotrophomonas* species are indicated on the tree leaves following an established Sm clade numbering (16, 18). The number of genomes/strains in collapsed clades is indicated. Former *Stenotrophomonas* species recently reassigned to the new genera *Parastenotrophomonas*, *Pseudostenotrophomonas*, and *Allostenotrophomonas* (18) are shown as collapsed clades. *Pedostrophomonas* genomes were excluded from the analysis. The scale bar represents the number of expected substitutions per site under the best-fit model LG4M+F+R4 chosen by IQ-Tree 2 according to the Bayesian information criterion.

The genome was 4,482,207 bp long, with an average coverage of 125× and a GC% of 66.6. Gene calling and genome annotation were performed using NCBI’s Prokaryotic Genome Annotation Pipeline (Docker image version 2023-10-03.build706) (14). The genome encodes 3,970 proteins, 24 pseudogenes, and 91 RNAs, including 4 rRNA operons. The gene encoding the chromosomal replication initiator protein DnaA was set as the first gene. Figure 1 shows a maximum likelihood species tree for the genus *Stenotrophomonas* inferred with IQ-TREE version 2.3.5 (15) from top-scoring core-genome proteins selected with GET_PHYLOMARKERS version 20230119 (16) and GET_HOMOLOGUES version 28082021 (17) from 103 input genomes, including relevant type strains (18). Sm18 grouped with the clinical *S. maltophilia* reference strains ATCC 13637^T^ and K279a. The Sm18 genome is valuable for studying the evolution of opportunistic pathogens in the environment and their interactions with free-living amoebae.

## Data Availability

The complete genome sequence of *Stenotrophomonas maltophilia* strain ESTM1D_MKCAZ16_6a (Sm18) and associated raw reads were deposited in GenBank under BioProject PRJNA1081934 with accession number CP146374.

## References

[1] Ochoa-Sánchez LE, Vinuesa P. 2017. Evolutionary genetic analysis uncovers multiple species with distinct habitat preferences and antibiotic resistance phenotypes in the Stenotrophomonas maltophilia complex. Front Microbiol 8:1548. doi:10.3389/fmicb.2017.01548 28861062 PMC5562727

[2] CLSI. 2016. Clinical and laboratory standards institute (CLSI) performance standards for antimicrobial susceptibility testing. 26th ed. Clinical and Laboratory Standards Institute, Wayne, PA.

[3] Argueta-Zepeda F-S, Rivera J, Valerdi-Negreros JC, Rensing C, Vinuesa P. 2025. The Stenotrophomonas maltophilia MntR miniregulon includes novel extracytoplasmic components and affects replication in Acanthamoeba castellanii phagosomes. bioRxiv. doi:10.1101/2025.10.14.682371

[4] Rivera J, Valerdi-Negreros JC, Vázquez-Enciso DM, Argueta-Zepeda F-S, Vinuesa P. 2024. Phylogenomic, structural, and cell biological analyses reveal that Stenotrophomonas maltophilia replicates in acidified Rab7A-positive vacuoles of Acanthamoeba castellanii. Microbiol Spectr 12:e0298823. doi:10.1128/spectrum.02988-23 38319117 PMC10913462

[5] Chen S, Zhou Y, Chen Y, Gu J. 2018. Fastp: an ultra-fast all-in-one FASTQ preprocessor. Bioinformatics 34:i884–i890. doi:10.1093/bioinformatics/bty560 30423086 PMC6129281

[6] Rrwick/Filtlong: Quality Filtering Tool for Long Reads. 2022. GitHub. Available from: https://github.com/rrwick/Filtlong

[7] Wick RR, Judd LM, Cerdeira LT, Hawkey J, Méric G, Vezina B, Wyres KL, Holt KE. 2021. Trycycler: consensus long-read assemblies for bacterial genomes. Genome Biol 22:266. doi:10.1186/s13059-021-02483-z 34521459 PMC8442456

[8] Li H. 2016. Minimap and miniasm: fast mapping and de novo assembly for noisy long sequences. Bioinformatics 32:2103–2110. doi:10.1093/bioinformatics/btw152 27153593 PMC4937194

[9] Wick R. 2024. Rrwick/Minipolish. Python. https://github.com/rrwick/Minipolish.

[10] Kolmogorov M, Yuan J, Lin Y, Pevzner PA. 2019. Assembly of long, error-prone reads using repeat graphs. Nat Biotechnol 37:540–546. doi:10.1038/s41587-019-0072-8 30936562

[11] Vaser R, Šikić M. 2021. Time- and memory-efficient genome assembly with Raven. Nat Comput Sci 1:332–336. doi:10.1038/s43588-021-00073-4 38217213

[12] Nanoporetech/medaka Python. 2024. Oxford Nanopore Technologies.

[13] Wick RR, Holt KE. 2022. Polypolish: short-read polishing of long-read bacterial genome assemblies. PLoS Comput Biol 18:e1009802. doi:10.1371/journal.pcbi.1009802 35073327 PMC8812927

[14] Tatusova T, DiCuccio M, Badretdin A, Chetvernin V, Nawrocki EP, Zaslavsky L, Lomsadze A, Pruitt KD, Borodovsky M, Ostell J. 2016. NCBI prokaryotic genome annotation pipeline. Nucleic Acids Res 44:6614–6624. doi:10.1093/nar/gkw569 27342282 PMC5001611

[15] Minh BQ, Schmidt HA, Chernomor O, Schrempf D, Woodhams MD, von Haeseler A, Lanfear R. 2020. IQ-TREE 2: new models and efficient methods for phylogenetic inference in the genomic era. Mol Biol Evol 37:1530–1534. doi:10.1093/molbev/msaa015 32011700 PMC7182206

[16] Vinuesa P, Ochoa-Sánchez LE, Contreras-Moreira B. 2018. GET_PHYLOMARKERS, a software package to select optimal orthologous clusters for phylogenomics and inferring pan-genome phylogenies, used for a critical geno-taxonomic revision of the genus Stenotrophomonas Front Microbiol 9:771. doi:10.3389/fmicb.2018.00771 29765358 PMC5938378

[17] Contreras-Moreira B, Vinuesa P. 2013. GET_HOMOLOGUES, a versatile software package for scalable and robust microbial pangenome analysis. Appl Environ Microbiol 79:7696–7701. doi:10.1128/AEM.02411-13 24096415 PMC3837814

[18] Chauviat A, Abrouk D, Brothier E, Muller D, Meyer T, Favre-Bonté S. 2025. Genomic and phylogenetic re-assessment of the genus stenotrophomonas: description of Stenotrophomonas thermophila sp. nov., and the proposal of Parastenotrophomonas gen. Nov., Pseudostenotrophomonas gen. Nov., Pedostenotrophomonas gen. Nov., and Allostenotrophomonas gen. Nov. Syst Appl Microbiol 48:126630. doi:10.1016/j.syapm.2025.126630 40550196

